# Revitalizing faith: an inquiry into political Sufism and religious continuity in contemporary Kazakhstan

**DOI:** 10.3389/fsoc.2024.1447966

**Published:** 2024-11-20

**Authors:** Nodar Karimov, Risalat-Bibi Karimova, Khalminyam Massimova, Gulzhakhan Khajiyeva

**Affiliations:** ^1^Center for Uyghur Studies, R.B. Suleimenov Institute of Oriental Studies of the Science Committee of the Ministry of Science and Higher Education of the Republic of Kazakhstan, Almaty, Kazakhstan; ^2^Higher School of Economics and Management Department, Turan University, Almaty, Kazakhstan

**Keywords:** political Sufism, Kazakhstan, Central Asia, folk Islam, syncretism, secularism, religious groups

## Abstract

The article embarks upon a study of political Sufism in contemporary Kazakhstan and patterns of religious continuity through an examination of Islamic manifestations that stretch back centuries, juxtaposed with the current state religious policy. It examines the role of the Muftiate’s “official Islam” in shaping the life of religious communities, exploring the intricate interplay between religious identity, secularism, and public perception in the post-Soviet landscape. The author employed a multi-faceted research approach, integrating historical analysis, policy examination, and an ethnographic study of contemporary religious dynamics in the country. The historical analysis provides a foundation of the religious landscape, while policy analysis examines the state’s contemporary role in shaping religious practices. Ethnography, focusing on groups such as the Jahriyya, Naqshbandiyya, and the Suhba, involves respondents from diverse regions of Kazakhstan, offering firsthand insights into the experiences and perceptions of Sufi communities at the grassroots level, which enriches the study with a contextual perspective. Notably, fieldwork surveys, where the author engaged with respondents, provide valuable insights into the diverse experiences shaping the dynamics of political Sufism and religious continuity. They involved a diverse group of respondents, including religious leaders, community members, and secular intellectuals, from multiple regions of Kazakhstan at both urban and rural levels. The article is of scientific and practical significance as it enhances understanding of religious dynamics in the country, offering policymakers, scholars, and practitioners valuable perspectives for informed decision-making and policy development. The research’s limitations include its focus on specific Sufi groups within Kazakhstan, which may not represent the full spectrum of Islamic practices across the region. The recommendations emphasize the need for further analysis of how prioritizing doctrinal adherence over individual freedoms contributes to the erosion of authentic religious institutions and the politicization of Islam. It also recommends to investigate the roles of Sufi groups in filling spiritual and social vacuums, their potential for politicization, and how this interplay affects religious freedom and national identity in Kazakhstan. This is particularly significant given the state’s efforts to appropriate Islamic traditions for ideological purposes, which has led to a separation between Islamic doctrine and its lived expressions. Finally, it emphasizes the need for further comparative research on Sufi movements across Central Asia to better understand how different state policies impact religious communities.

## Introduction

The article embarks upon a study of political Sufism in contemporary Kazakhstan and patterns of religious continuity through an examination of Islamic manifestations that stretch back centuries, juxtaposed with the current state religious policy. It examines the role of the Muftiate’s “official Islam”^1^ in shaping the life of religious communities, exploring the intricate interplay between religious identity, secularism, and public perception in the post-Soviet landscape. The article opens with a brief historical account of the characteristics of Islam in Kazakhstan. It contends that Islamic identity of the country has been historically determined by multicultural rather than a monoreligious trend and displayed itself through quasi-state models of political organisation, community/governance networks, multiethnic and multireligious societies, individual freedoms and syncretic essence of Islam in the local culture. It also reflects on transformative effects on Kazakh Islam of the Soviet-era religious and social policies. Shaped by Soviet-era secularization policies, the current religious structures reflect a legacy of regulated Islam, where religious education and practices were limited to state-sanctioned institutions. This historical backdrop is essential for understanding the dynamics of modern religious practices, which continue to be influenced by Soviet attempts to institutionalize and control Islamic traditions. The next section deals with contemporary disputes surrounding the influence of “traditional Islam” and “foreign influences” on the religion. It explores conflicts among state-sanctioned ethnic Islam, Sufis, conservative Hanafites, fundamentalist Salafis, and reformist movements, shedding light on theological disagreements and the political implications of these tensions. The article then proceeds to discuss the multifaceted resurgence of Sufism in post-Soviet Kazakhstan, examining its complex interplay with politics and the evolving dynamics within various Sufi groups. It highlights the state’s attempts to “legalize” Sufism by incorporating it into the framework of state-sanctioned ethnic Islam, emphasizing figures like Ahmad Yasavi as symbols of “traditional Islam.” Yasavi is portrayed not only as a spiritual figure but also as a national symbol aligned with state interests. By framing Yasavi’s teachings as integral to the nation’s cultural heritage, the state effectively uses Sufism to maintain social cohesion, align religious practices with its ideological goals, and counter competing Islamic movements. The section delves into the activities, challenges, and diverse compositions of notable Sufi groups such as Jahriyya, Naqshbandiyya, and the emergent “Suhba” group, shedding light on their distinctive ideologies, political implications, and the evolving landscape of Sufi movements in the country. In conclusion, the article questions the state’s attempt to appropriate Islamic traditions for ideological purposes, resulting in the rise of informal ultra-conservative preachers and the potential for increased politicization of Sufi groups, emphasizing their role in the re-Islamicization of the population and the challenges posed by their evolving ideologies and political ambitions.

## Methods

The author employed a multi-faceted research approach, integrating historical analysis, policy examination, and an ethnographic study of contemporary religious dynamics in the country. These approaches are systematically integrated by first establishing a historical foundation of Islamic practices in Kazakhstan, followed by an analysis of how state policies shape current religious dynamics, and culminating in an ethnographic exploration of Sufi communities to understand their contemporary experiences. Each approach informs the other, creating a cohesive framework that allows for a comprehensive understanding of political Sufism in Kazakhstan. Application of narrative literature review broadened understanding within a specific field (Greenhalgh et al., 2018) and emphasized significant findings about the subject (Nielsen and Einarsen, 2018). A historical analysis was conducted to understand the evolution of Islamic practices and the impact of Soviet-era policies on religious life in Kazakhstan. It examines sources that explore medieval Islam and Sufism in Central Asia, followed by analyses of Soviet-era policies and contemporary ethnographic accounts. Key references include Levshin’s (1832) detailed account of Kazakh culture and religious practices, Olcott’s (2007) exploration of medieval Sufi orders, Privratsky’s (2001) work on the evolution of Kazakh Islam, Khalid’s (2007) analysis of post-Soviet Islamic dynamics, Hismatullin’s (2001) study of Sufism in Central Asia, Babadzhanov’s (2009) research on Sufi rituals and typologies in Central Asia, and Muminov’s (2018) study of Sufi groups in Kazakhstan. This combination of historical texts and modern scholarship provides a comprehensive perspective on how different historical periods have shaped political Sufism in Kazakhstan.

The selection of literature was based on the relevance to key themes such as the historical evolution of Sufism, state-religion relations, and the socio-political dynamics of religious identity in Central Asia, ensuring that sources provide a well-rounded understanding of political Sufism in Kazakhstan. Preference was given to peer-reviewed journals, monographs, and primary sources that offer critical insights into the intersection of religious practices and political frameworks, with a focus on works that present empirical evidence, historical context, or theoretical contributions directly related to the study’s objectives.

This was complemented by a detailed examination of contemporary state policies and their influence on the religious landscape. The policy analysis included reviewing governmental and Muftiate documents, legal texts, and public statements to contextualize the state’s approach to managing religious diversity and Sufi practices specifically.

To achieve the objectives of the research the author applied the principles of Bourdieu (1977) “practice theory,” which suggests that the foundations of collective identity are in the daily behavior of those who ascribe to that identity. Using the “practice theory,” the author: (1) traced a historical perspective of Islam in Central Asia; (2) discovered relationship between Sufis and “official Islam” and how those patterns played out in the state dynamics and group consciousness; (3) analyzed religious life of Sufi communities of the region; analyzed how political Sufism navigates political constraints and asserts its role as both a religious and political force; (4) analyzed Islam and identity in the modern period.

The ethnographic studies involved a diverse group of respondents, including religious leaders (such as mosque imams and “informal imams”), regular Muslims who attend “official mosques” and participate in religious classes but do not identify as Sufis or Salafis, Sufi and Salafi community members, and secular intellectuals from multiple regions of Kazakhstan, covering both urban and rural areas. The sample included approximately 30 participants, selected using purposive, convenience, and snowball sampling methods to ensure representation across different Sufi (Jahriyya, Naqshbandiyya, and Suhba) and Salafi groups. Demographic profiles varied by age, gender, education, and occupation, providing a broad understanding of the social composition within these communities. This provided valuable insights into the diverse experiences shaping the dynamics of political Sufism and religious continuity.

To ensure the validity of the methods, triangulation was employed, integrating historical analysis, policy examination, and ethnographic fieldwork. This combination allowed for cross-verification of findings across different sources, enhancing the credibility of the results.

The reliability of the study was maintained through consistent application of data collection procedures, standardized interview protocols, and regular respondent validation, ensuring that the interpretations accurately reflect participants’ perspectives and historical contexts.

The article is of scientific and practical significance as it enhances understanding of religious dynamics in the country, offering policymakers, scholars, and practitioners valuable perspectives for informed decision-making and policy development.

## A historical perspective of Islam in Central Asia

Among features characterizing Islam in Central Asia is its inherent diversity, which, over centuries, has interwoven with the aspirations of local communities and engaged in a continuous dialogue with national cultures. Historically, this region has been witness to a rich array of Islamic manifestations, encompassing diverse movements such as Sunni and Shiite factions (Murji’ah, Isma’ilism, and Mubayida), Sunni theological schools (Maturidism, Ash’arism, and Karramiyyah), Sunni schools of jurisprudence (Hanafism and Shafiism), and Sufi orders (Naqshbandiyya, Yasaviyya, Kubraviyya, Ishkiyya, Qadiriyya, and Afaqiyya). Islam has thrived through these multifaceted forms engaging with local national cultures, which resulted in the emergence of a distinctive Central Asian form of Islam that remains an integral part of the global Islamic civilization.

Such intricate mosaic has shaped the syncretic, flexible and liberal^2^ nature of Islam in the region. In the nomadic steppes this pattern manifested itself through quasi-state models of political organization, community/governance networks, multiethnic and multireligious societies, individual freedoms, free trade system, and syncretic essence of Islam in the local culture. Analyzing Kazakh hordes of the sixteenth and seventeenth centuries Martha Olcott defines them as “federations or unions of self-governed tribes” that “bear the mark of military democracy rather than a feudal society” (Olcott, 1987, p. 10–11). Beyond this, tribes and clans acted as strong institutions of nomadic civil society effectively limiting the power of khan and protecting ordinary people from the tyranny of the authorities (Sabitov, 2015). Alexey Levshin describes elections and intensive debates over the nomination of khan as one of the most important ceremonies in the political life of the Kazakh people (Levshin, 1832, p. 126–8). Each Kazakh bii (judge) had his own court, which he supported by collecting 10% of all fines levied (Levshin, 1832, p. 14–5). Within the larger Kazakh community, the clan leaders and elders had far more influence than the khan. They allocated lands to the auls (villages) and families, they had control of the warriors, and, ulike the khan, they had an unquestioned right of taxation as their right to part of each family’s herd (Levshin, 1832, p. 15). No khan could, without reason, punish or kill a member of any Kazakh tribe, since the latter was under protection of his tribe members—distant and close relatives. Zhaksylyk Sabitov cites an example of Zhiembet-zhirau, a prominent commander and batyr (“hero”) of the Kazakh Younger Zhuz (“horde”), who together with his brother threatened to raise his tribe against Ishim Khan because of his systematic abuse of power. Even the Kazakh riots against the policy of the Russian Empire were, for the most part, driven by particular tribes’ desire to defend their tribal lands (Sabitov, 2015).

## Sufis and folk Islam in Central Asia

For centuries, Sufi governance networks incarnated decentralized system and influenced the complexity of ways Islam operated and evolved in Central Asia. Here, the evolution of Islam did not occur through monoreligious trend and linear progression via centralized system with communities, branching the mosaic of highly syncretic and distinct cults, deities, saints, orders, and ideas. Sufi communities consolidated the protection of private property and run their own communal funds and trusts. They effectively transmitted wealth across generations through the creation of the charitable foundation, the waqf, which operated independently from the state. They managed personal status issues, like marriage, death, and inheritance.

Sufis have always been largely autonomous and ideologically distinctive from the orthodox Hanafi ulama. Karen Armstrong sees in Sufism a reaction against the growth of jurisprudence or Sunni mainstream schools of law, which seemed to some Muslims to be reducing Islam to a set of purely exterior rules (Armstrong, 2002, p. 74). In the teachings of the Yasaviyya order, which is attributed to the famous Central Asian Sufi Khoja Ahmad Yasavi, one would find a deep synthesis of Arab and Turkic cultures, Tengrianism^3^ and Islam. Yasavi order has preserved the elements of Tengrianism as a symbol of the philosophy of life of the Turks (Ayupov, 2004, pp. 278–9). Sufism preserved the basic ideas and rites of Shamanism, the personalities of the shaman and the Sufi are so merged that shamans are included in the ranks of Muslim saints (Orynbekov, 2005, p. 129). Yasavi made the city of Yasi into the major centre of Islamic learning for the Kazakh Steppe (Basri, 2020). His shrine was a site of strong spiritual and historic importance during the period of the Kazakh Khanate (1465–1847), especially for the khans of the Middle Horde, because burial there was viewed as interment in sacred ground (Olcott, 2007, p. 3). The Yasaviyya order performed a complex function of transmitting pre-Islamic folk traditions, values, ideological orientations aligning them to the dominant Islamic culture.

However, it was political dimension of Sufism that played a central role in the history of Central Asia. Sufi leaders helped define relations between the ruler and the ruled during the time of Timurid rule in fourteenth-sixteenth century. Naqshbandiyya without doubt has been the dominant Sufi brotherhood in Central Asia. Effective intercommunal relations, political, religious, and cultural autonomy developed by Naqshbandi sheikhs led to serious suspicions on the rulers’ side. For example, during the meeting of Baha ud-din Naqshband with Malik Husain, the ruler of Herat, the latter began questioning the shaykh about his dervish^4^ (whether it was hereditary or not), his practice of zikr^5^ (whether he practiced loud zikr), sama’ (musical ceremonies), and halvat (hermitary). Identification with institutional Sufism spoke of its impact on communal life and was perceived by the rulers as a threat to centralized political power, which complicated the relationships of Sufi shaykhs with the rulers. In response to hostility from the political authorities, Naqshbandiyya tried to appeal to several types of legitimizing principles that fluctuate from hereditary continuity, with its strict discipline and spirituality, to “silsila”^6^ ties that later became decisive for most Sufi communities (Hismatullin, 2001, p. 233) and group solidarity. Naqshbandiyya solidarity was always central for the brotherhood and was even higher than the bonds of Islam. Maulana Muhammad-Qazi, one of the main disciples of the prominent Sufi leader Khoja Akhrar quoted his teacher: “If I learned about the *kafir*^7^ in China, who speak highly of the outstanding (shaikhs) of this ta’if^8^ and their words, I would enter into his company without hesitation and without condemning him for being a *kafir*” (Hismatullin, 2001, p. 185). These legitimizing principles have always played significant role in the competitive political struggle between the Sufi groups and the authorities in Central Asia.

Principles of communal self-governance applied by Naqshbandis deserve particular attention. Naqshbandis have ideologically shifted from such a traditional element of political life in Central Asia as “hereditary transfer of power.” Their sources are full of derogatory and outlawing references to the shaykhs of Mirasi, mostly linked to Yasaviyya tradition, who were “trading in their father’s shops” (Hismatullin, 2001, p. 187). Among the fiercest shaykhs of Naqshbandiyya was Maulana Chusti, who had a stake in defining political affairs in the Ferghana Valley and Samarkand in 16th century. Chusti and his followers were hostile to the Shaikhs of Kubraviya and other rival Sufi groups intensely antipathizing the “hereditary shaykhs.” One such “hereditary rival” was a charismatic shaykh Abd al-Qadir Turbati, who came with his supporters to Maulana Chusti to win over him. Instead, he was struck by a plague and soon “went to rest” at the grave of his ancestors (Hismatullin, 2001, p. 187). Chusti himself broke with the principle of hereditary succession by refusing to follow in the footsteps of his father Mawlana Fathallah and joining Yasaviyya in favor of Naqshbandiyya Khojjagan (Hismatullin, 2001, p. 187).

Members of the Sufi orders had individual responsibilities towards their communities (quasi-states), but at the same time, they had freedom in political and ideological self-identification. The change in political views and preferences could at times involve not only individual shaykhs or members of the order, but the whole groups or communities. A vivid example of it is conversion of Khwarazm Yasaviyya community into Nashbandiya ideology in the early 17th century. Tokum Shaykh Khivaki of Yasaviyya order brought his 400 murids to the Shaykh of Naqshbandiyya and thereby “transferred” them to new brotherhood (Hismatullin, 2001, p. 238). Here, we observe a mature Sufi shaykh with a fair number of followers passing on to another shaykh, which simultaneously indicate a collective shift and the degree of individual freedoms in the community. This case also reflects modern examples where communities adapt their identities or beliefs due to social or political influences, a process that continues to impact collective identities.

All modern Naqshbandiyya sheikhs in Central Asia begin the chain of their spiritual succession with Khoja Ahrar. Khoja Ahrar’s religious justification for politicization of the brotherhood’s activity was clear: “One should go to the rulers having raised the religion of the prophets to its limits, so that their throne and crown appeared insignificant compared to the eminence of the faith.” He was highly critical of elevating *adat* law (customary law) to be the law of the state, superseding Islamic laws. He was also a strong critic of the ruling elite that imposed additional taxes—secular taxes not based on the Sharia—on the people. Khoja Ahrar’s biographers suggest that in his relations with rulers he most often used the technique of pacification from the position of force, supported by his own economic might and the might of his supporters as well as his authority as a spiritual leader (Olcott, 2007, p. 7). Khoja Ahrar also introduced a new ethical norm that stipulated that the sheikh and members of the brotherhood could and should be wealthy although he warned that they should have “their hearts not tied to their wealth.” This peculiar capitalism with an ethical twist gave impetus to the economic activity of the brotherhood. From then on, many sheikhs of the brotherhood were among the wealthiest people of Transoxiana (Olcott, 2007, p. 8).

Sufis were a source of legitimization for the rulers of the eighteenth century khanates in the region as well as a source of mobilizing protest during the last decades of Russian colonial rule (Olcott, 2007, p. 2). Sufism of the Yasaviyya order, and, subsequently, of the Naqshbandiyya order, was the incarnation of Islam in the pre-colonial period. Then Sufis acted as political subjects, rather than objects of complex political, social and cultural transformations.

Religiosity of ordinary Muslims was mainly expressed through observing the rules of everyday, or folk Islam, intertwined with Sufism. Folk Islam was characterized by a mixture of native animistic beliefs with Islamic traditions in the popular consciousness. These practices and beliefs rely upon traditional magic and rituals that call upon the supernatural world. Folk Islam appeared to be more tangible for the grasroots communities rather than formal and complicated doctrines of the orthodox Islam, which was comprehensible mostly to ulama (Islamic scholars) who possessed religious education. Kazakhs professed a particularly archaic form of folk Islam. Alexey Levshin, depicting the life in the nomad steppes in the beginning of the nineteenth century, has indicated that the Kazakhs “in general had a notion of the highest being who created the world, but some worshipped him according to the laws of the Qur’an, others mixed the teachings of Islam with the remnants of ancient idolatry, the third believed that apart from the deity of the good, who cared about the happiness of people and whom they called “Kudai,” there is an evil spirit, or Shaitan, the source of evil’ (Levshin, 1996, p. 313). The Kazakhs have long preserved the religious rites of their pre-Islamic ancestors. Adapting religion to ethnic culture, the official and unofficial religious establishment absorbed many local cults that shaped religious consciousness of the people giving it syncretic and polystructural character (Toleubaev, 1972, p. 6).

## Islam and identity in the modern period: secular challenges and religious minimalism

The Soviet era had deep and far-reaching consequences in Muslim Central Asia. Its transformative effects first emerged in the administrative setup and governance, and soon spread to the religious and social domains. Very soon the region lost its connection with the Muslim world. Bolsheviks set course for secularisation of Islam attempting to adapt it to the ideas of Marxism and Leninism in order to make communist ideology attractive to the peoples of the Muslim East. The creation of religious administrations—the Muftiates—in the post-war period provided authorities with the means to achieve this goal by reaching believers and influencing their religious practices. Multiple factors might have been behind the decision to create religious administrations, but the bottom line was the creation of an “official Islam” in the USSR that was used to frame religious practices as well as an instrument of foreign policy when dealing with Muslim countries.

The public practice of religion and religious teaching was kept under the tight control of the Soviet state. From 1948 to 1991, there were only two functioning medreses/madrasahs (Islamic schools) in the whole of Central Asia: the Mir-i Arab medrese opened in Bukhara in 1946 and the Al-Bukhari medrese in Tashkent founded in 1956. In 1971, the Institute for Higher Islamic Studies was established in Tashkent. Private religious education was banned making it impossible for the ordinary Muslims to learn the basics of their religion legally. This has backfired increasing anxiety and protests among the population and gave rise to the informal religious study cells, or hujrah, which were an open secret, winked at by the KGB (Olcott, 2007, p. 30). These, alongside a network of underground Sufi orders, became referred to as “parallel Islam,” which co-existed with “official Islam,” promoted and controlled by the Muftiate.

After the demise of the Soviet Union, Islam started again gaining popularity in Kazakhstan. It has become an important source of identity for the people, serving both as an escape from everyday problems and a channel to express the distrust in the existing political and social processes. The number of practicing believers has been on rise: by 2020 they already made up 14% of Kazakhstan’s Muslims. In some regions, such as Mangistau, this figure went up to 37% (Natsionalniy doklad, 2020, pp. 83–84). Kazakhstani leaders are keenly aware that Islam is an important cultural marker that distinguishes natives from Slavic settlers. It is hardly a surprise therefore that the government uses, among other ideologies, the symbols of Islam to build new national identity.

Concurrently, the public space remains de-Islamized and profoundly secular. In the words of Adeeb Khalid, there remains a substantial residue of the Soviet suspicion of religion and its potential to be put to unhealthy political uses (Khalid, 2007, p. 121). The state make use of this suspicion in its attempts to control Islam. However, it does not do so in a vacuum, for the suspicion is widely shared by large sections of the population. In the Soviet era, public claims could only be made with reference to universal human values seen in materialist terms. Religion was considered a human construct, and a harmful one at that. That situation has not changed substantially. Islam is not God’s binding command to humanity but an aspect of human creation. Sufism (and Islam in general) are thus being judged by external criteria (Khalid, 2007, p. 121).

At the grassroots community level a sense of pride in Islam as part of the national heritage can coexist with complete lack of religious observance or any belief, let alone a desire to live in an Islamic state. Anthropologist Bruce Privratsky coined the term “religious minimalism” to describe this attitude. During his field research in Kazakhstan, he found that most people characterize their religious affiliations as musilmanshiliq, literally “Muslimness,” or taza jol, “the clean path,” rather than “Islam.” Privratsky notes that “this reflects discomfort with the abstraction of Islam as an ideology and a preference for Muslim life as an experience of the community.” According to Privratsky, being Muslim for the majority of Kazakhs is connected to the reverence of saints and holy places. Saints are perceived as guardians of the taza jol for the community, while holy places, including shrines and mosques, imbue the very land on which Kazakhs live as Muslim. This religious minimalism does not mean, however, that Central Asians do not see themselves as Muslims; rather, it means that they see Islam as an integral part of their way of life (Privratsky, 2001).

## Navigating “traditional” and “foreign” Islam

The contemporary disputes in Kazakhstan involving both state and non-state entities revolve around the interplay of “traditional Islam” and “foreign influences” on Islam. These disputes unfold between the adherents of the state-sanctioned ethnic (folk) Islam, Sufis, conservative Hanafites, fundamentalist Salafis, and certain reformist movements. The work of the Muftiate could often spark outrage in the wider society, where allegations of “Arabization” of Kazakh folk Islam are emerging (Mustafina, 2020). It is no coincidence that “Nur-Mubarak” university, which actively cooperates with the Muftiate, is known for “Arabization” of its Islamic training and maintaining exchange programmes with Egyptian universities, such as Al-Azhar (Mostovoi, 2018). Arabized Islamic training of the Muftiate could possibly lead to homogenization, erosion of “Kazakh traditional Islam” and its transformation from a syncretic to a puritanical one, which puts greater emphasis on rituals and codes of conduct than on substance.

Another example is growing stand-off between the Muftiate and pro-Salafi Muslims outside the Muftiate. It is best exemplified by the Muftiate-run online religious forums, which the author analyzed as part of his field research between 2015 and 2017. Tensions arise not merely over observance of universal Islamic principles and rituals, but also over profound theological issues, such as aqidah (the Islamic creed) and al-asma wa al-sifaat (Allah’s names and attributes). Discussions between the official imams moderating the forum and their followers on one side and the Salafis on the other have often been controversial accompanied by verbal abuse and a rhetoric of intolerance. Although the politicization of theological issues indicates increased fragmentation of religious community, it is likely to be exploited for certain political objectives.

Salafis, for their part, strongly condemn Sufi teachings and practices such as the veneration of shaykhs, the visitation of shrines and other sacred sites, etc. These features are regarded by fundamentalists as polytheism (shirk) and innovation (bidʿa). Salafis call themselves representatives of “traditional Islam” or “orthodox Islam.” They regard the Sufis as contradicting the oneness of God (tawḥid) and “pure Islam.” Local Salafis are supported by many foreign charities and organizations in their fight against Sufi groups, such as “Istilah,” “Tayba,” and the “Committee of the Muslims of Asia.” (Wilkowsky, 2009).

## Political Sufism in contemporary Kazakhstan

As posited by the renowned Islamic scholar Adeeb Khalid, Post-Soviet Sufism is not a return to the past but the creation of something new (Khalid, 2007, p. 120). Likewise, the intersection of Sufism and politics in Kazakhstan initiates complex dynamics, presenting potential political risks. As the government grapples with sustaining political stability and social cohesion in a diverse society, the role and influence of Sufi orders in shaping public sentiment cannot be overlooked.

The resurgence of Sufism in the region is evident in the increased frequency of pilgrimages to sacred sites. The veneration of saints, a significant aspect of Islamic practice among Kazakhs, not only experiences a revival but also achieves peak popularity among diverse segments of the population. Shrines like the mausoleums of Khoja Akhmad Yasavi, Arystan-baba, Ukasha-ata, Beket-ata, Shopan-ata, Karaman-ata mosques, and the graves of ancestral saints like Yrgyzbai-ata, Bayanbai-ata, Domalak-ana, Baidibek-ata, among others, attract mass pilgrimages. The number of shrines and their devotees is steadily increasing. Restoration and renovation efforts extend not only to well-known Kazakh shrines but also to forgotten graves of ancestors, transforming them into places of mass pilgrimage. At nearly every shrine, pilgrims encounter guidelines emphasizing the observance of ritual purity and essential Muslim practices, including reading namaz, fasting, and more.

Understanding historical role Sufi communities have played in the formation of the “Kazakh Islam” the state is attempting to «legalize» Sufism by placing it under the umbrella of the state sanctioned ethnic Islam. For example, the twelfth-century Sufi leader and poet, Ahmad Yasavi is regarded by the Kazakh government as a “Kazakh national saint,” and the founder of “traditional Islam.” It is unclear what role Yasaviyya Sufi legacy plays in this ideology, but Ashirbek Muminov points to other, less official evocations of this ideology on a national scale. On an expedition to Kostanay, Northern Kazakhstan, and Akmola in 2008, he recorded stories from local informants. Some of these stories described individuals referred to as “piradar” who obtained that title by making a pilgrimage to the shrine of Yasavi, in southern Kazakhstan, and being initiated by Sufi shaykhs on their trip (Muminov, 2018, p. 296). There is also a case of neighboring Uzbekistan, where Sufism was initially viewed by the government not only as the spiritual heritage and the foundation for national revival, but also as an aspect of Islam able to resist political Islamic ideologies, such as Wahhabism, Salafism, and Hizb al-Tahrir. However, after the 600th anniversary of the birth of Hoja Aḥrar, the official attitude toward Sufism began to change. During this anniversary, the political roles of past Sufis such as Hoja Aḥrar were publicized, and the Uzbekistani authorities became wary of Sufism (Babadzhanov, 2001, pp. 25–30).

Contemporary Sufis continues to spawn their own culture and distinctiveness from the orthodox Hanafi clergy and exhibits all the characteristics of an independent religious-political movement. In the words of modern Sufi leaders, a tariqah is to the science of tasawwuf what a mazhab is to the science of fiqh. It is a means to arrive at Allah’s pleasure in regards to matters of the heart, and, just like the mazhabs in fiqh, tariqahs are grounded in the Qur’an and the Hadiths of the Prophet (Nabawi, 2015). This is likely one of the reasons why Sufism in Kazakhstan is not presently permitted as full-fledged ideology and a functioning religion. Rather it is limited to a set of purely exterior rules, cultural rudiment and folklore. Only within these limitations the Muftiate is allowed to preach about Sufism. Southern Kazakhstan is informally regarded as the main breeding ground for religious extremism in the country. The region was inhabited by sedentary agricultural population earlier than other regions, consequently establishing deep-seated roots for Islam in the region. The Southern city of Turkistan is the birthplace of Kazakh Islam and home to the mausoleum of Ahmad Yasavi, founder of the Sufi order Yasaviyya. In 2004, the Spiritual Administration of Muslims of Kazakhstan (SAMK) tested imams’ theological knowledge in the region. In an effort to identify people with possible subversive links, officials asked imams which madhab they prefer; any other answer than Hanafi school would suggest that the cleric may have been influenced by radicals (Karagiannis, 2010).

But despite the barriers, Sufism experiences a revival at the institutional level, with emerging circles coalescing around charismatic leaders. Perhaps the most notable example is the community of Kazakh Sufis, the Jahriyya, led by Sheikh Ismatullah. Bakhtiyar Babajanov attributes the revival of the Yasaviyya-Qadiriyya ritual tradition in post-Soviet Kazakhstan exclusively to his leadership. Sheikh Ismatullah, originally from the Kazakh diaspora in Afghanistan, resided in Pakistan before eventually settling in Kazakhstan. Today, he has numerous followers in cities across Kazakhstan and Kyrgyzstan. His community is known for practicing collective ritual involving a combination of three or four types of “dhikr jahr” (loud remembrance of God) (Babadzhanov, 2009, p. 115).

The recent field research, conducted by the author, suggests that the group’s composition is diverse and reflects a broad cross-section of society, highlighting the inclusive nature of the membership. The group includes entrepreneurs, day laborers, social activists, the unemployed, students, pensioners, and civil servants, as well as many others. Intellectuals, encompassing both religious scholars and educated secular specialists, predominantly young and middle-aged individuals, are also part of the group. This cohort include media professionals, young scientists, graduate students, employees of various companies, and university faculty, who convincingly assert their Kazakh and Muslim identity while gaining a deeper understanding of the ethnic customs and culture. They view Sufism as a pure Islam and an authentic layer of their national culture, untainted by Russian influence. A distinct group includes girls and women for whom it’s a journey of self-discovery and a means to find like-minded individuals and a supportive community.

In the late 1990s, the Jahriyya group was banned because it operated mosques and madrasas but was not registered with the Muftiate. However, in the early 2000s, the group resumed its activities in Almaty and later expanded across the country. When the group registered a public association called “Senim. Bilim. Omir” (“Belief, Knowledge, Life”), it had 17 branches and 16 offices in Kazakhstan. The main bases of the group are in the cities of Astana, Almaty, Uralsk, and Turkistan (Muminov, 2018, p. 287). In 2005, Sheikh Ismatullah released the book titled “The ABC of Quranic Sciences,” leading the Muftiate to issue a fatwa declaring his doctrine harmful to Muslims and contrary to the Sharia. In 2011 following a closed trial, nine leaders of the Jahriiya group, including Sheikh Ismatulla, were sentenced to long term imprisonment. The lawyers defending the Sufi group spoke of the politically motivated and unreasonably harsh charges (Toguzbayev, 2011).

In 2019, Sheikh Ismatullah was released from prison, and very soon positive narratives about his mission, his revered ancestors, and his challenging fate began surfacing on the internet, being promoted by opinion leaders, including social activists, scholars, and entrepreneurs. In 2020, Sheikh even released an official address to the presidents of Kazakhstan, Turkey, Russia, and the United States of America, urging the widespread practice of dhikr. But what’s particularly noteworthy is that, during Sheikh’s imprisonment, his followers carried on with the organization’s activities, attracting new adherents, pursuing education abroad, publishing books about the group’s teachings for a diverse audience, and actively utilizing social networks to spread the message. Assylbek Izbairov points to political risks associated with the Jahriyya group and warns against the resurgence of political Sufism in the country, drawing parallels with the transformation of Naqshbandiyya Sufi group ideology into neo-Wahhabi (Izbairov, 2008:22).

The Naqshbandiyya is believed to be the second largest Sufi group in Kazakhstan. The group is mainly presented in the Southern Kazakhstan with its main center located in Uzbekistan at the mausoleum of Bahauddin Naqshband. Southern Kazakhstan is characterized by a significant Uzbek population, comprising both indigenous residents and migrant workers. It engages in political activities and leverages social networks to disseminate its teachings. The group has its own management system, with the qalpe (khalifa) at its helm. “Khalifas” run the regional organizations of this Sufi group. In Turkistan district, the khalifa and leader of the organization is Nasir al-Din Ishan Abduvoitov. The qalpe of Kazakhstan is Qurban-Ali Akhmetov. There are additional members of this group throughout Central Asia, mainly in Uzbekistan (Muminov, 2018, p. 285). The Naqshbandi often considered by Kazakhstani authorities to be “Uzbek” or “foreign”: the commissioner for religious affairs in the South Kazakhstan oblast argues that the followers of this group are Uzbeks and Tajiks, for which reason they are viewed with suspicion and thought to represent possibly problematical “Uzbek” influences (Muminov, 2018, p. 293).

New to the country’s Sufi landscape is the “Suhba” group established in 2015 by its spiritual leader Murat Hakim. Educated at Egypt’s al-Azhar University and with experience in secular institutions, Hakim advocates for using Russian in sermons to disseminate his teachings, given the limited proficiency in Kazakh among the local intelligentsia and youth (Temirbayeva et al., 2022). Notably, this goes against the Muftiate attempts to “nationalise” Islam through “special” language policy, which largely ignores the Russian-speaking Muslims. At present, many of them cannot interpret the content of the Kazakh-language Friday sermons (Mavloniy, 2010) and do not attend the preachings by imams because of the language barrier. Rather, they receive religious guidance and other information from alternative sources, such as the Internet, books and audio lectures. This is likely an example of how artificial barriers to “folk” Islam prompt the Muslim population to turn to more adaptable Russian-speaking Islamic preachers. With its own organizational structure, the “Suhba” group is reaching out to all strata of society, prioritizing civil servants, businessmen, social activists, and scientists, and actively involving women in leadership roles.

The remaining Sufi groups in contemporary Kazakhstan, include the Kadiri, “Kenesary Sarbazdary” and a number of modern Turkish groups. Each group has its authentic ideology and structure. The Kadiri group is monoethnic and is exclusively prevalent among Chechens and Ingush. The major mosque of the Chechens in Astana is named after Shaykh Kunta Ḥajji. Many Chechens, and above all the followers of Vis Ḥajji Zagiev, live together in the villages of Krasnaia Poliana, Arbuzinka, and Petrovka, located around Astana, the capital of Kazakhstan. They follow the religious practice of Vis Ḥajji, who is also called the “Atbasar Shaykh,” because he began his preaching in the environs of the town of Atbasar. Vis Ḥajji’s grave is located in Arbuzinka, and is a place of pilgrimage for his murids. His immediate master (ustaz) was Dada Aḥmad, who received his training from Shaykh Chim-mirza, a successor of Kunta Ḥajji. In the 1950s, during the exile in Kazakhstan, Vis Ḥajji informed the murids of Chim-mirza that he had established direct contact with the teacher, Kunta Ḥajji in the spiritual world, and that Kunta Ḥajji had revealed to him the true meaning of his teaching, entrusting him with the mission of preaching the “pure path.” Kadiriya group is seen as closed to outsiders due to its cautious attitude toward state authorities (Muminov, 2018, p. 293).

Members of the Sufi group “Kenesary Sarbazdars” align themselves with the Shaziliyya tariqa, following the teachings of the Egyptian Sheikh Yusri Rushti. The group’s leader, Ergali Kopeev, claims descent from a saint (auliyya) in the northern region of Kazakhstan, identifying himself as an ishan—one of the titles of Sufi tariqat leaders. Ideologically, the group embraces pan-Turkic perspectives, advocating for the unification of Turkic peoples and the revival of the Turanian army. They actively comment various events in public life both in Kazakhstan and internationally, while also engaging in fundraising activities (Temirbayeva et al., 2022).

The main activities of Turkish Sufi groups are in the educational sphere. They do not emphasize silsilas in their teaching and communicate through modern mass media, particularly print media. They have also opened schools, which again do not emphasize the transmission of knowledge from individual master to individual student, but from teachers—in the modern sense—to students. They have opened 28 high schools as well as two Kazakh-Turkish universities: The University of Foreign Languages and Business, and Suleyman Demirel University in Almaty (Muminov, 2018, p. 287). Despite Turkish groups outwardly striving to demonstrate their non-political stance, their presence in the education sector and involvement with children and youth pose an inherent risk. It carries mobilization potential and serves as a tool for soft power, promoting the interests of a foreign state within the country’s borders.

## Conclusion

Division of Islam into “good” folk and “bad” non-traditional and recruitment of the clergy not only heightens apparent contradiction between officially sanctioned and actual religious practices but is leading to politicisation of religion. Sufism is no exception, as evident in the confrontation between the Jahriyya group and the Kazakhstani state. The state does not uniformly seek to eliminate Islam from society, but it attempts to appropriate Islamic traditions for ideological ends of the nation-state, which results in separation and isolation of Islamic doctrine from its lived aspects. The doctrinal dimension is prioritized over personal and religious freedoms, leading to the eradication of authentic religious institutions and the condemnation of certain practices as ignorant social behavior.

The research revealed that the spiritual and social needs of local communities, once met by traditional communal institutions, are now overlooked, creating a vacuum actively filled by ultra-conservative preachers who often socialize believers to view radical organizations as legitimate. Muslims more and more turn for moral guidance to informal leaders and seek authority and leadership outside state religious structures, which open up on the one hand possibilities for the state to fill the gap and on the other for the radicals to step in and legitimize violent protest. In essence, politicization of Islam stem from the securitization of religion, communal deprivation and absence of coherent national ideology.

The implications of religious securitization in Kazakhstan manifest through the state’s intensified efforts to monitor and control religious expression, resulting in a climate of distrust and fear of potential repercussions among communities. This increased surveillance not only stifles legitimate religious practices but also alienates individuals who may have otherwise engaged positively with their faith. Moreover, the framing of religious beliefs as security threats can lead to what Fathali Moghaddam attributes to “communal deprivation” which has high probability of catalyzing religious extremism (Moghaddam, 2011, p. 49).

Sufi groups in Kazakhstan already offer examples of direct political involvement and the relationship between the Sufis and the state is not stable. Given the state’s awareness of the historical political role of Sufism, it is unlikely to permit the emergence of alternative Islamic authority in the country. It means, under certain circumstances, the Sufi groups could become more politicized. While Sufi groups currently play their role as “liberal Muslims,” revivers of “Kazakh Islam,” and counterbalances to Islamic fundamentalism, Sufism represents a religious organization with a powerful ideology and an effective and closed rank structure, which have seemingly enabled Sufis groups to be the sole survivors of the Soviet era in Central Asia. Sufism is characterized by the orthodoxy of its followers and a commitment to reform, while healthy competition and individual mobility within Sufi groups cultivate strong leaders.

The Kazakhstani state has yet to define its relationship with Sufi groups, but it has already encountered the need to consider factors such as the political component in the activities of Sufi groups, the trajectory of transformation in Sufi ideology and practices, the extent of rivalry and cooperation among different Sufi movements, opposition to Sufism by Salafis, and the perception of Sufism by non-Sufis.

Sufis have already demonstrated their potential as significant drivers of the re-Islamicization of the population. The secular intelligentsia is increasingly drawn towards Sufism and actively seek membership in Sufi brotherhoods. This increases the likelihood of Sufi politicization due to the political ambitions of the intelligentsia Naqshbandiyya and Qadiriyya Sufi groups played this role earlier being politicized brotherhoods in the Caucasus, Central Asia, Turkey, and Kashgar since the fifteenth century. Overall, the reawakening of historical memory, especially among the new generation of Sufi leaders, can become a significant driver of politicization. As expressed by one Naqshbandi leader, the distinguishing feature of Naqshbandiyya is the fact that they “always cut the roots of tyrants and rulers (Olcott, 2007, p. 38).

In light of these findings, several concrete recommendations for policymakers are proposed. Firstly, researching how the imposition of doctrinal Islam weakens authentic religious institutions and politicizes Islam is essential. Secondly, examining the roles of Sufi groups in addressing the spiritual and social needs of the population, their potential for politicization, and the implications of this interplay for religious freedom and national identity in Kazakhstan is crucial. This examination is particularly important given the state’s efforts to appropriate Islamic traditions for ideological ends, resulting in a disconnect between Islamic doctrine and its practical expressions. Finally, further comparative research on Sufi movements across Central Asia is necessary to better understand how varying state policies influence religious communities.

## Footnotes

^1^The term is used to articulate the state institution, officially titled the *Spiritual Administration of Muslims of Kazakhstan*, overseeing religious policy in the country. Along with the Committee for Religious Affairs of the Ministry of Culture and Information of Kazakhstan, this body plays a key role in defining and promoting the state-approved version of Islam, shaping the religious landscape by controlling religious groups and their activities.

^2^Favourable to or respectful of individual rights and freedoms (Oxford Dictionaries: https://en.oxforddictionaries.com/definition/liberal).

^3^Ancient belief system originating among the Turkic and Mongolic peoples of Central Asia. It centers on the worship of Tengri, the sky god, and incorporates elements of shamanism and animism. Tengrism teaches that humans should live in harmony with nature and the universe, viewing all existence as interconnected.

^4^Member of a Sufi Muslim religious order known for their ascetic lifestyle and spiritual devotion. In some Sufi orders spiritual leadership or membership may be passed down through generations. This hereditary aspect often pertains to the role of the shaykh (spiritual leader) within the order, who may pass on the leadership to a family member. Also in many Sufi traditions, when a dervish (disciple) takes the hand of the shaykh (spiritual guide), it symbolizes a deep spiritual connection. This act, known as baya’ah or the oath of allegiance, is considered a means of transmitting spiritual light, wisdom, and blessings from the shaykh to the disciple. This connection is believed to link the disciple to the spiritual lineage of the order, making them a vessel for the Divine Light and allowing them to receive and transmit spiritual insights that flow through the chain of transmission, or silsilah, back to the Prophet Muhammad.

^5^Sufi practice of the remembrance of God. This can involve repeating sacred phrases, names of God, or specific prayers. Zikr can be practiced either silently or aloud. The purpose of zikr is to cultivate a closer connection to the Divine, purify the heart, and keep God constantly in one’s thoughts.

^6^Spiritual lineage or chain of transmission within Sufism. It represents the unbroken line of spiritual mentorship that connects a Sufi shaykh (teacher) back through previous teachers to the Prophet Muhammad. Each Sufi order has its own silsila, which is considered a source of spiritual authority and legitimacy.

^7^Refers to a non-Muslim or disbeliever. In Islamic terminology, “kafir” traditionally denotes someone who does not accept the Islamic faith.

^8^Refers to a group, sect, or community, specifically the Sufi brotherhood or order to which the speaker belongs. The term “ta’if” can denote a collective of people with shared beliefs or practices.
